# Successful Use of Cyclophosphamide as an Add-On Therapy for Multiple Myeloma Patients with Acquired Resistance to Bortezomib or Lenalidomide

**DOI:** 10.1155/2013/651902

**Published:** 2013-03-28

**Authors:** Shigeki Ito, Tatsuo Oyake, Kazunori Murai, Yoji Ishida

**Affiliations:** Hematology & Oncology, Department of Internal Medicine, Iwate Medical University School of Medicine, Morioka, Iwate 020-8505, Japan

## Abstract

Novel agents such as thalidomide, lenalidomide, and bortezomib have been shown to possess potent activity against multiple myeloma. However, the treatment strategy for patients who acquired resistance to these agents has not been established. In addition to switching drug classes, intensified treatment strategy, including increase in the dosage of current agents and addition of other agents, may be considered for these patients. We here describe 2 myeloma patients with acquired resistance to bortezomib or lenalidomide, in whom add-on therapy with low-dose cyclophosphamide was effective and tolerable. These cases suggest that add-on therapy with cyclophosphamide is one of the treatment options to overcome resistance to novel agents in patients with multiple myeloma. A larger prospective study is needed to clarify the efficacy and safety of this strategy for novel agent-resistant multiple myeloma.

## 1. Introduction

Introduction of immunomodulatory drugs (IMiDs), such as thalidomide and lenalidomide, and the proteasome inhibitor bortezomib has dramatically improved outcomes in patients with multiple myeloma (MM) [1–4]. However, MM remains incurable, with most patients responding to therapy but eventually relapsing. Approximately 50%–60% of patients with relapsed or refractory MM (RRMM) and receiving each novel agent in combination with dexamethasone had a partial or better response [5–8]. When further relapse developed in patients who received novel agents, treatment with other drug classes or with the same agents is indicated. Intensified treatment with conventional cytotoxic drugs plus novel agents is also an attractive treatment strategy for RRMM. Bortezomib has been shown to restore both melphalan and doxorubicin sensitivity in resistant cell lines and synergize with melphalan in killing myeloma cells [9, 10]. Thalidomide and lenalidomide have also been shown to enhance the efficacy of various antimyeloma agents [11–13].

Alkylating agents have been the standard of care for myeloma patients for many years. A series of clinical trials in the UK in the 1980s demonstrated the equivalent efficiency of cyclophosphamide (CY) to melphalan and highlighted its favorable toxicity profile, with less profound myelosuppression and lack of stem cell toxicity, compared to melphalan [14]. In addition, CY has been studied in combination with lenalidomide or bortezomib for relapsed disease, with excellent efficacy and safety [15–18]. However, it remains uncertain whether add-on therapy with CY is effective for MM patients who progressed or relapsed during treatment with novel agents. Here, we report for the first time 2 cases in which the use of low-dose CY as an add-on therapy was effective and tolerable in MM patients who developed resistance during lenalidomide or bortezomib treatment. 

## 2. Case Report

**Case 1**

**Figure 1:**
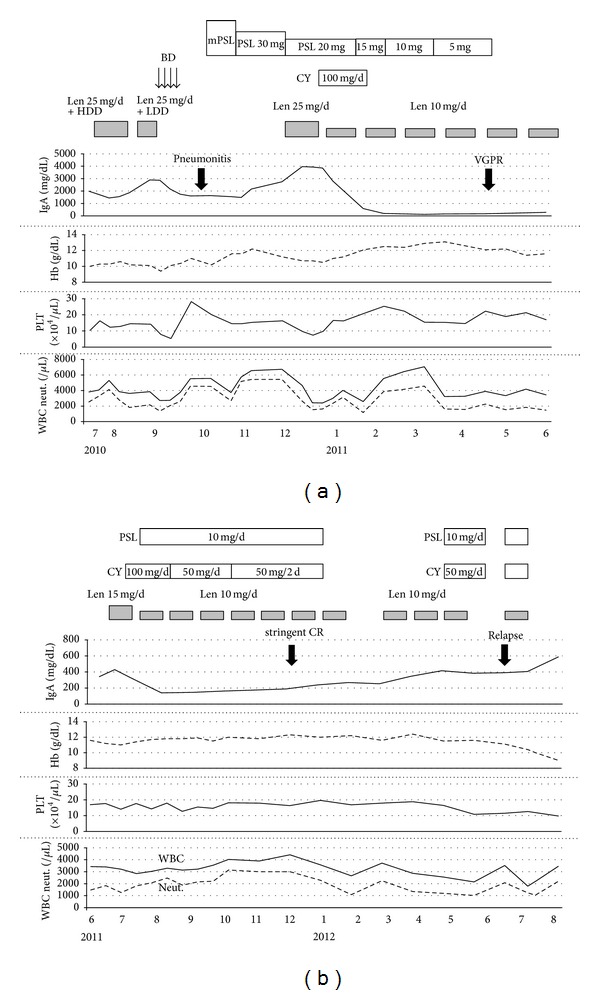
Clinical course of Case 1. mPSL: methylprednisolone; Len: lenalidomide; HDD: high-dose dexamethasone; LDD: low-dose dexamethasone; BD: bortezomib plus dexamethasone; CY: cyclophosphamide; Hb: hemoglobin; PLT: platelet counts; WBC: white blood cell counts; and neut.: neutrophil count.

A 69-year-old man was diagnosed with symptomatic MM immunoglobulin (Ig) A lambda in February 2008. He received melphalan and prednisone (MP) regimen. As the therapy was ineffective, a combination of vincristine, doxorubicin, and high-dose dexamethasone (VAD) followed by vincristine, melphalan, ranimustine, and high-dose dexamethasone regimen was administered since April 2008, after which he achieved partial response (PR) according to the International Myeloma Working Group uniform response criteria [19]. In June 2009, he developed progressive disease (PD), and a combination of bortezomib and dexamethasone (BD) was administered. He then achieved complete response (CR) after 2 cycles; a weekly bortezomib maintenance therapy was then administered. In December 2009, he again developed relapse during the maintenance therapy and received 7 cycles of bortezomib, melphalan, and prednisone (BMP) regimen. However, no better response was obtained and peripheral neuropathy (grade 2) was developed. Therefore, the patient received lenalidomide (25 mg/day for 21 days every 28-day cycle) and high-dose dexamethasone (40 mg/day, days 1–4, 9–12, and 17–20 every 28-day cycle) in July 2010. The clinical course is shown in Figure 1. Because he developed PD after 2 cycles of the therapy, retreatment with twice-weekly BD regimen was administered; however, interstitial pneumonia with hypoxia developed. He was admitted to our hospital and received steroid pulse therapy. During tapering prednisone (PSL), IgA level was elevated despite administration of lenalidomide (25 mg/day for 21 days every 28-day cycle). Under the administration of PSL (20 mg/day) and low-dose lenalidomide (10 mg/day for 21 days every 28-day cycle), continuous low-dose CY (100 mg/day) was added. After the first cycle of therapy, IgA level decreased to the normal range. Administration of CY and PSL was interrupted because mild epigastric discomfort developed. After 5 cycles of lenalidomide monotherapy, IgA level was elevated again. The add-on therapy with CY and PSL to lenalidomide was resumed, and the patient achieved stringent CR in December 2011. The add-on therapy was discontinued in January 2012 followed by low-dose lenalidomide monotherapy. In February, he developed grade 3 neutropenia. The treatment was withheld, and 1 month later, the neutrophil count was recovered. He then received 2 additional cycles of lenalidomide monotherapy and 2 cycles of add-on therapy, but as the disease progressed in July 2012, he discontinued the add-on therapy. No other severe adverse event has been observed.

**Case 2**

**Figure 2:**
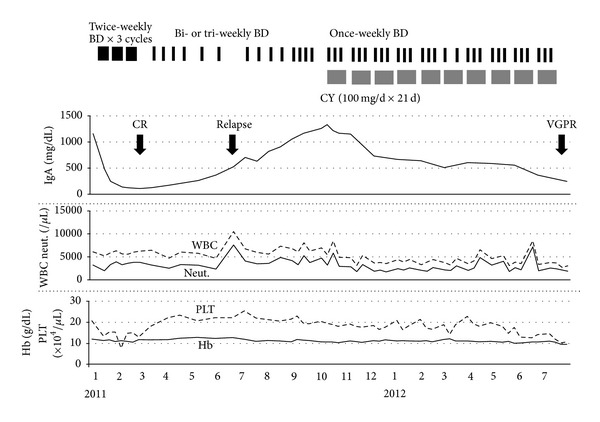
Clinical course of Case 2. BD: bortezomib plus dexamethasone; CY: cyclophosphamide; Hb: hemoglobin; PLT: platelet counts; WBC: white blood cell counts; and neut.: neutrophil count.

A 53-year-old man was diagnosed with symptomatic MM IgA lambda in February 2007. He received VAD regimen as an induction therapy. After harvesting peripheral blood stem cells following conditioning with high-dose CY, he received high-dose melphalan followed by tandem autologous stem cell transplantation (ASCT) in December 2008 and July 2009, resulting in CR. However, he developed relapse in October 2010. The clinical course is shown in Figure 2. He received BD regimen as salvage therapy and achieved CR after 3 cycles; a maintenance therapy with bi- or tri-weekly bortezomib alone was administered. In July 2011, he developed relapse again and received once-weekly BD regimen since September 2011; however, the disease progressed. Therefore, he was treated with the addition of low-dose CY (100 mg/d, days 1–21 every 28-day cycle) to the weekly BD regimen. After 10 cycles of add-on therapy, he achieved very good PR (VGPR). No severe adverse event has been observed.

## 3. Discussion

The addition of CY to thalidomide (CTD (CY, thalidomide and dexamethasone)) [20, 21], lenalidomide (CRD (CY, lenalidomide and dexamethasone), REP (lenalidomide, CY and PSL)) [15, 16, 22], or bortezomib (CyBorD (CY, bortezomib and dexamethasone)) [17, 18, 23] has been shown to increase better response rates and possibly prolong survival of patients with newly diagnosed MM or RRMM. Thus, the addition of CY enhances the therapeutic effects of novel agents in myeloma patients. However, it is unknown whether the addition of CY overcomes the resistance in patients who had novel agent-refractory myeloma. We presented here that add-on therapy with CY was effective and tolerable in patients with MM who developed resistance to lenalidomide or bortezomib.

In the first case, the patient was considered to have lenalidomide-refractory MM. van de Donk et al. reported the efficacy of combination regimen with lenalidomide, CY, and PSL for patients with RRMM, including lenalidomide-refractory patients [16]. The regimen consisted of lenalidomide (10 mg/day for 21 days, every 28-day cycle), combined with continuous low-dose CY (100 mg/day) and PSL (10–20 mg/day). All patients were heavily pretreated and had received a median of 6 previous lines of antimyeloma therapy. Thalidomide and bortezomib were previously administered to most patients, and all patients had received alkylating agents along with lenalidomide plus dexamethasone. Response to therapy was observed in 64.3% of patients, including 14.3% with CR, 21.4% with VGPR, and 14.3% with PR. Interestingly, treatment with the regimen resulted in CR in 2 patients who progressed during treatment with lenalidomide plus dexamethasone. In our case, low-dose CY was added to reduce the doses of lenalidomide and PSL. Our patient had received 6 previous lines of antimyeloma therapy. A remarkable response was observed during the first cycle. Although the administration of CY was discontinued because of gastric discomfort, the patient achieved VGPR during low-dose lenalidomide plus PSL therapy. Because IgA level increased during lenalidomide monotherapy, CY and PSL were added to lenalidomide again. The patient achieved stringent CR after 5 cycles of the therapy. Thus, lenalidomide in combination with CY and PSL has clinical activities in both heavily pretreated patients and in lenalidomide-refractory myeloma. Clinical studies with lenalidomide in combination with CY and dexamethasone or PSL showed that neutropenia was the most relevant and frequent side effect. Morgan et al. reported the efficacy and safety of lenalidomide (25 mg/day for 21 days every 28-day cycle) in combination with CY (500 mg/day, days 1, 8, 15, and 21 every 28-day cycle) and dexamethasone (40 mg/day, days 1–4 and 12–15 every 28-day cycle) for RRMM. In the report, grade 4 neutropenia was observed in 8 (38%) of 21 patients [22]. Schey et al. reported the results of a Phase I/II dose escalation study to determine the maximum tolerated dose (MTD) of CY when combined with lenalidomide and dexamethasone in RRMM. The MTD was 600 mg CY on days 1 and 8. Grade 3/4 hematological complications occurred in 26% of patients, grade 3/4 infections in 3%, with thromboembolic complications in 6% [15]. van de Donk et al. reported that grade 3/4 neutropenia was observed in 43% of patients and grade 3 infection and deep venous thrombosis were observed in 21% and 14%, respectively [16]. Although our patient had mild gastric discomfort and grade 3 neutropenia, the side effects were resolved when CY or lenalidomide was withheld. Thus, the addition of CY to low-dose lenalidomide and PSL seems to be safe and manageable. However, the optimal dose of CY as an add-on therapy has not yet been defined. Further prospective clinical study is needed to clarify this issue.

In the second case, the patient received treatment with BD regimen for relapse 2 years after tandem ASCT. Although CR was obtained, the patient relapsed during BD maintenance therapy. Because the patient had PD during treatment with once-weekly BD regimen (bortezomib 1.3 mg/m^2^ as a single bolus i.v. on days 1, 8, and 15 every 28-day cycle, dexamethasone 20 mg/day once daily on the day of bortezomib injection, and the day thereafter), oral CY (100 mg/day for 21 days every 28-day cycle) was added. After 10 cycles of therapy, the patient achieved VGPR. No severe adverse events were observed. This case indicated that addition of low-dose CY is effective and safe in patients who progressed during BD therapy. Kropff et al. reported a Phase II trial of bortezomib in combination with intermediate dose of dexamethasone, and continuous low dose of oral CY has shown encouraging antimyeloma activity for relapsed MM [23]. In the study, patients received eight 3-week treatment cycles with bortezomib 1.3 mg/m^2^ on days 1, 4, 8, and 11, followed by three 5-week cycles with bortezomib 1.3 mg/m^2^ on days 1, 8, 15, and 22. Within all cycles, dexamethasone (20 mg/day) was administered orally on the day of bortezomib injection and the day thereafter. In addition, patients received CY continuous oral treatment at a dose of 50 mg/day once daily. Response to therapy was seen in 82% of patients, including 16% with CR and 66% with PR. On the other hand, adverse events of grade 3/4 in at least 10% of patients comprised leucopenia, infection, herpes zoster, thrombocytopenia, neuropathy, and fatigue. In particular, grade 3/4 thrombocytopenia and peripheral neuropathy were seen in 53% and 21% of patients, respectively. Our patient had no severe hematological and nonhematological adverse events; may be a result of our low-dose schedule of bortezomib (4-week cycles with bortezomib 1.3 mg/m^2^ on days 1, 8, and 15 every 28-day cycle).

These cases suggest that add-on therapy with CY is one of the treatment options to overcome resistance to novel agents in patients with MM. Larger prospective study is needed to clarify the efficacy and safety of this strategy for novel agent-resistant MM.
